# The risk of kidney transplant graft loss in sensitized vs. unsensitized patients is modified by prior transplant status

**DOI:** 10.3389/frtra.2026.1712618

**Published:** 2026-02-16

**Authors:** A. Nicholson, K. Tennankore, A. J. Vinson

**Affiliations:** 1 Dalhousie Faculty of Medicine, Medical School, Halifax, NS, Canada; 2 Division of Nephrology, Department of Medicine, Nova Scotia Health, Halifax, NS, Canada; 3 Kidney Research Institute Nova Scotia, Halifax, NS, Canada

**Keywords:** antibodies, kidney transplant, panel reactive antibody, prior transplant, rejection, risk prediction, sensitization

## Abstract

**Background:**

A prior kidney transplant (KT) has been associated with an increased risk of graft loss following repeat transplantation. This study examined the risk of adverse posttransplant outcomes between patients with and without a prior transplant, depending on sensitization status.

**Methods:**

We used propensity score matching to examine the combined exposure of sensitization status [panel-reactive antibody (PRA) 0%, >0%–80%, and >80%] and first/repeat KT as a nested variable in adults across the US [2000–2017; Scientific Registry of Transplant Recipients (SRTR)]. We then used multivariable Cox and logistic regression models to examine the association between the nested variable and death-censored graft loss (DCGL), all-cause graft loss (ACGL), and delayed graft function (DGF). Effect modification between PRA status (20%) and prior KT status was assessed for each outcome.

**Results:**

Among 38,660 matched patients, DCGL (adjusted Hazard Ratio (aHR) 1.56, 95% CI 1.47–1.66), ACGL (aHR 1.42, 95% CI 1.35–1.49), and DGF (adjusted Odds Ratio (aOR) 1.89, 95% CI 1.75–2.03) risk was highest with a prior KT and PRA >80% (vs. unsensitized transplant-naïve patients). The risk associated with increased PRA was greater in those with a prior transplant. Prior KT modified the association between increased PRA and DCGL, ACGL, and DGF (*p*-value < 0.001).

**Conclusion:**

Prior KT status modified the association between PRA and adverse outcomes. Sensitized patients (PRA >80%) with a prior KT faced higher risks of DCGL, ACGL, and DGF compared with sensitized patients without a prior KT.

## Introduction

Although kidney transplantation significantly improves both quality of life and patient survival, graft loss is an important consequence with negative downstream repercussions (1, 2). In 2020, 13% of patients added to the kidney transplant waitlist in the United States had a prior transplant—a statistic that has increased over time (3, 4). In fact, between 1996 and 2005, there was a 40% increase in the absolute number of repeat kidney transplantations in the United States (5). The causes of graft loss are multifactorial and include both T-cell-mediated and antibody-mediated rejection (TCMR and AMR, respectively) (6). Patients with preformed antihuman leukocyte antigen (HLA) antibodies are at increased risk of posttransplant AMR, even in the absence of donor-specific antibodies (DSA) (7). Higher panel-reactive antibody (PRA) levels, or sensitization status, not only increase the risk of graft loss but also limit access to later kidney transplantation (8). While desensitization strategies are becoming more commonly utilized, crossing pre-existing DSA is still not the standard of care in most programs (9, 10). Patients may become sensitized through various mechanisms, including blood transfusions, pregnancy, and previous kidney transplantation (11–14). While prior kidney transplantation is a leading cause of sensitization, not all patients with a prior kidney transplant develop detectable anti-HLA antibodies (15). Non-HLA antibodies are also known to result in increased rejection risk but are not measured as part of standard pretransplant work-up (16). Therefore, patients with a prior kidney transplant who remain unsensitized to HLA antigens may still have undetected non-HLA antibodies that could impact subsequent allograft survival (17). Whether unsensitized patients with a prior transplant are at increased risk for adverse graft outcomes—and how this compares to sensitized transplant-naïve patients—remains unclear. Given the known association between a higher PRA and subsequent transplant rejection, PRA (but not prior transplant status) is utilized by many programs to dictate decision-making regarding induction immunosuppression (18). However, if prior transplant status increases the risk of rejection and/or graft loss, irrespective of sensitization status, then perhaps antecedent transplant history should likewise be considered in the immunologic risk profile of transplant candidates, independent of their PRA. Therefore, the aim of this study was to examine the relative risk of adverse posttransplant outcomes [death-censored graft loss (DCGL), all-cause graft loss (ACGL), and delayed graft function (DGF)] associated with sensitization status in those with vs. without a prior kidney transplant.

## Methods

### Sample population

We conducted a cohort study of adult patients (≥18 years of age) who underwent a first or second living or deceased donor kidney transplant in the United States (U.S.) between 1 January 2000, and 31 December 2016, identified using the Scientific Registry of Transplant Recipients (SRTR). We excluded patients who were <18 years of age, those with >1 prior transplant or a missing transplant number, those with missing values for PRA, those who had undergone ABO-incompatible transplant, and those who had received multiple organs, en-bloc, or sequential transplants. Patients undergoing repeat transplant whose first transplant occurred at <18 years of age were not excluded.

### Exposure

We examined the combined exposure of sensitization status (PRA 0%, >0%–80%, and >80%) and prior kidney transplant status by creating a nested variable with six categories: (i) no prior transplant, PRA 0%; (ii) no prior transplant, PRA >0%–80%; (iii) no prior transplant, PRA >80%; (iv) prior transplant, PRA 0%; (v) prior transplant, PRA >0%–80%; and (vi) prior transplant, PRA >80%. The reference category was no prior transplant, PRA 0%. PRA was defined as the peak cumulative PRA prior to either first or repeat kidney transplant.

### Outcomes

The primary outcome was DCGL, defined as either return to dialysis or pre-emptive re-transplant. Secondary outcomes included ACGL (defined as a composite outcome of death or graft loss) and DGF (defined as need for dialysis in the first week after transplant).

### Data collection and covariates

Known predictors of DCGL were collected including HLA match status, preemptive recipient status, donor–recipient weight ratio, donor and recipient demographics (age, race, sex), recipient comorbidities (hypertension, coronary artery disease, peripheral vascular disease, diabetes mellitus), type of initial kidney disease (glomerulonephritis, diabetes, polycystic kidney disease, hypertension, other), transplant year, recipient transplant center, and donor type (living, deceased).

### Statistical analyses

Baseline characteristics were described after stratification by prior kidney transplant status. Continuous variables were summarized as means and standard deviations for normally distributed data and medians with interquartile ranges for non-normally distributed data. Categorical variables were presented as counts and percentages. Significant differences between groups were determined using *t*-tests, Wilcoxon rank-sum tests, or chi square tests, as appropriate.

### Primary analysis

Given differences in age and comorbidity burden between transplant-naïve and repeat transplant recipients, we performed a propensity score (PS)-matched analysis (using logistic regression with 1:1 matching for prior kidney transplant status without replacement, and caliper width 0.001) to create better balance for patient factors between groups, creating as comparable populations as possible. Patients with and without a prior kidney transplant were matched based on nearest-neighbor matching for donor and recipient race, sex, age, donor–recipient weight ratio, HLA match status, recipient coronary artery disease, peripheral vascular disease, hypertension and diabetes, cause of recipient end-stage kidney disease (ESKD), preemptive transplant status, transplant year, recipient transplant center, and donor type, but not PRA.

In this matched population, we used Cox proportional hazards models to examine the association between our nested prior transplant–PRA variable with time to DCGL. Because the cohort was matched for the variables listed previously, we did not readjust for them in a multivariable model. Time to DCGL was depicted using Kaplan–Meier survival curves for the nested prior transplant–PRA variable in the matched sample.

### Secondary analyses

In a secondary analysis, we used Cox proportional hazards models in the matched cohort to examine time to all-cause graft loss (ACGL). Finally, we used logistic regression in the matched cohort to examine the adjusted odds of delayed graft function for our nested variable.

For all primary and secondary analyses, we reported the number at risk, number of events, proportion of patients with an event, risk ratio point estimates and exact 95% confidence intervals, *p*-values, and incidence rates per 100 person-years.

### Effect modification

In the matched cohort, we examined whether having a prior kidney transplant modified the known association between PRA (defined as 0%–20%, >20%) and each outcome (DCGL, ACGL, or DGF). This was assessed by including an interaction term between prior kidney transplant status and PRA in respective Cox proportional hazards and logistic regression models. This PRA cut point was selected based on prior literature (19, 20).

### Sensitivity analyses

We performed the following sensitivity analyses:

We repeated our primary and secondary analyses after redefining our nested variable to include more granular PRA cut points [(i) no prior transplant, PRA 0%; (ii) no prior transplant, PRA >0%–20%; (iii) no prior transplant, PRA >20%–80%; (iv) no prior transplant, PRA >80%; (v) prior transplant, PRA 0%; (vi) prior transplant, PRA >0%–20%; (vii) prior transplant, PRA >20%–80%; and (viii) prior transplant, PRA >80%]. The reference category was no prior transplant, PRA 0%.
In the PS-matched cohort, we examined the risk of each of DCGL, ACGL, and DGF associated with prior kidney transplant (vs. transplant naïve) after stratifying by PRA at time of transplant: 0%, >0%–80%, >80%. We adjusted these stratified models for PRA as a continuous variable.
Instead of PS matching, we conducted multivariable Cox proportional hazards and multivariable logistic regression analyses in the entire cohort, adjusting for the covariates listed previously.
We examined the odds of transplant rejection (acute T-cell-mediated or antibody-mediated) using a logistic regression model in the PS-matched cohort among patients with available rejection data (missing in 73.9%).
Finally, after PS matching, we examined the median warm ischemia time (WIT) in those with and without a prior transplant, and adjusted models for WIT (the time from organ removal from cold storage to reperfusion with warm blood) as per prior literature (21, 22). WIT was not included in our primary analysis on account of a high degree of missingness (35.3% missing).

All statistical analyses were performed using Stata version 14.2 (Stata Corp., College Station, TX, USA).

## Results

After relevant exclusions, the final cohort included 220,045 kidney transplant recipients, of whom 27,044 (12%) had a prior kidney transplant. Cohort derivation is shown in Supplementary Figure S1. Overall, 108,351 patients were unsensitized with a PRA of 0%. Of these, 103,178 (95.2%) were transplant-naïve and 5,173 (4.8%) had a prior kidney transplant. Baseline characteristics stratified by prior kidney transplant status are presented in Table 1. Among transplant-naïve patients, the median PRA was 0% (Q1 0, Q3 13), whereas the median PRA was 57% (Q1 5, Q3 92) for those with a prior transplant. The median age in those without and those with a prior kidney transplant was 52 (Q1 42, Q3 56) and 45 (Q1 35, Q3 54) years, respectively. Comorbidities such as diabetes, polycystic kidney disease, and hypertension were higher in those without prior kidney transplant.

**Table 1:** Baseline characteristics from overall cohort stratified by prior kidney transplant status.

Variables	No prior kidney transplant, *N* = 193,001	Prior kidney transplant, *N* = 27,044	*P*-value
Donor sex			<0.001
Male	100,847 (52.3%)	14,675 (54.3%)	
Female	92,154 (47.8%)	12,369 (45.7%)	
Recipient sex			<0.001
Male	117,732 (61.0%)	15,798 (58.4%)	
Female	75,269 (39.0%)	11,246 (41.6%)	
Donor age	41 (28, 51)	38 (25, 48)	<0.001
Recipient age	52 (42, 61)	45 (35, 54)	<0.001
Donor race			<0.001
Black	25,690 (13.3%)	3,092 (11.4%)	
White	159,900 (82.9%)	23,146 (85.6%)	
Other	7,381 (3.8%)	803 (3.0%)	
Recipient race			<0.001
Black	49,897 (25.9%)	5,753 (21.3%)	
White	129,531 (67.1%)	20,014 (74.0%)	
Other	13,570 (7.0%)	1,277 (4.7%)	
Preemptive			<0.001
Yes	35,623 (18.5%)	4,187 (15.5%)	
No	155,603 (80.6%)	22,576 (83.5%)	
HLA			<0.001
0	16,100 (8.3%)	4,187 (15.5%)	
1	7,049 (3.7%)	1,450 (5.4%)	
2	17,342 (9.0%)	2,595 (9.6%)	
3	35,249 (18.7%)	4,879 (18.0%)	
4	41,221 (21.4%)	5,707 (21.1%)	
5	49,325 (25.6%)	5,686 (21.0%)	
6	26,065 (13.5%)	2,443 (9.0%)	
Recipient comorbidities			
Diabetes	62,263 (32.3%)	5,371 (19.9%)	<0.001
Hypertension	155,313 (80.5%)	20,593 (76.2%)	<0.001
PVD	9,158 (4.8%)	1,039 (3.8%)	<0.001
CAD	15,663 (8.1%)	1,655 (6.1%)	<0.001
Cause of ESKD			<0.001
Diabetes	49,492 (25.6%)	3,282 (12.1%)	
GN	45,021 (23.3%)	9,445 (34.9%)	
PCKD	19,523 (10.1%)	1,578 (5.8%)	
Hypertension	47,651 (24.7%)	4,340 (16.1%)	
Hereditary	3,104 (1.6%)	1,416 (4.4%)	
Drugs	4,281 (2.2%)	300 (1.1%)	
Other	17,720 (9.2%)	3,978 (14.7%)	
Donor type			<0.001
Deceased	120,116 (62.2%)	18,321 (67.7%)	
Living	72,885 (37.8)	8,723 (32.2%)	
Donor BMI			<0.001
≤18.49	7,091 (3.7%)	1,149 (4.3%)	
>18.49–24.99	67,504 (35.0%)	10,095 (37.3%)	
>24.99–29.99	65,524 (34.0%)	8,900 (32.9%)	
>29.99–34.99	31,599 (16.4%)	4,105 (15.2%)	
>34.99	15,980 (8.3%)	2,156 (8.0%)	
Recipient BMI			<0.001
≤18.49	4,461 (2.3%)	1,084 (4.0%)	
>18.49–24.99	55,032 (28.5%)	10,722 (39.8%)	
>24.99–29.99	60,524 (31.4%)	7,569 (28.0%)	
>29.99–34.99	39,138 (20.3%)	3,706 (13.7%)	
>34.99	19,339 (10.0%)	1,661 (6.1%)	
Donor–recipient weight ratio (kg)			<0.001
<10	59,326 (30.7%)	8,140 (30.1%)	
10 to 30	37,791 (19.6%)	6,556 (24.2%)	
>30	18,858 (9.8%)	3,816 (14.1%)	
−10 to −30	45,717 (23.7%)	5,238 (19.4%)	
<–30	26,933 (14.0%)	2,703 (10.0%)	
Peak PRA	0 (0,13)	57 (5,92)	<0.001
0	103,178 (53.0%)	5,173 (19.1%)	<0.001
>0–80%	78,047 (40.4%)	11,954 (44.2%)	
>80%	11,776 (6.1%)	9,917 (36.7%)	
Year of transplant	2008 (2004, 2011)	2007 (2004, 2011)	

HLA, human leukocyte antigen; PVD, peripheral vascular disease; CAD, coronary artery disease; ESKD, end-stage kidney disease; GN, glomerulonephritis; PCKD, polycystic kidney disease; BMI, body mass index; PRA, panel reactive antibody.

Missing data: Donor race (0.01%), recipient race (0.00%), preemptive (0.93%), HLA (0.34%), recipient diabetes (1.33%), recipient hypertension (4.34%), ESKD (4.17%), donor BMI (2.70%), recipient BMI (7.62%), donor-recipient weight ratio (2.26%), recipient PVD (4.54%).

### Primary analysis

After propensity score matching, the cohort consisted of 19,330 patients with and 19,330 patients without a prior kidney transplant. There was good balance in the matched sample with no significant differences in any of the matched variables (mean standardized differences ≤3.8% for all). Baseline characteristics of the propensity score-matched population are presented in Supplementary Table S1.

**Figure 1:**
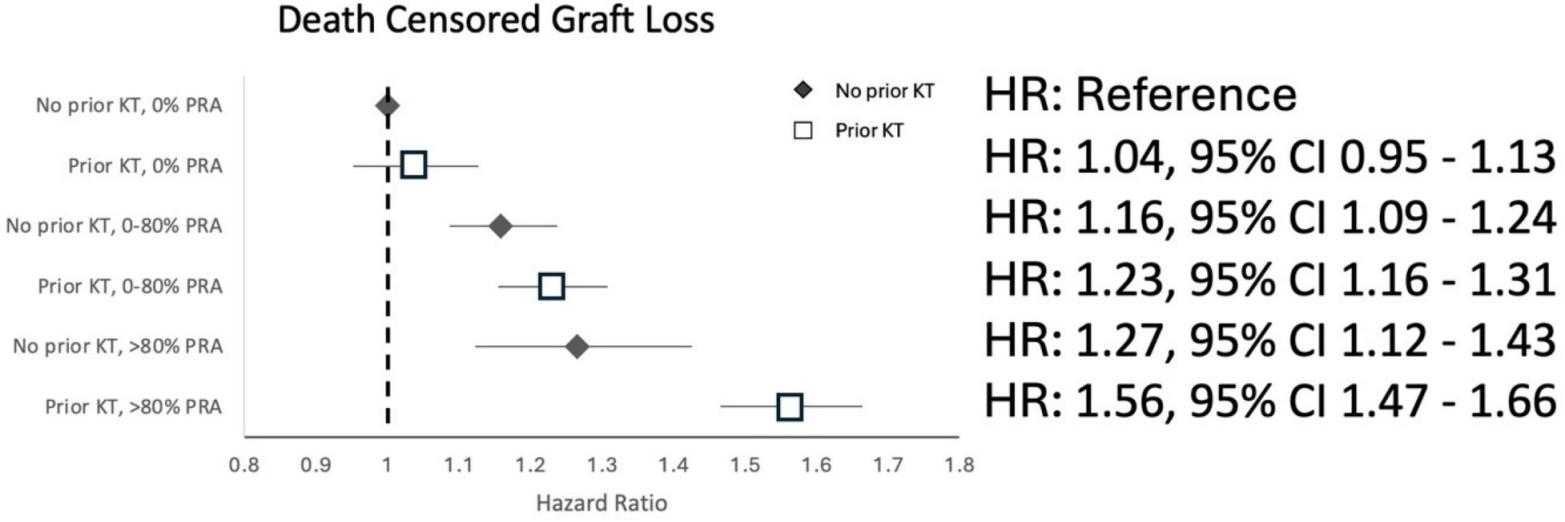
Hazard ratios for death-censored graft loss associated with combined prior transplant and PRA categories in the propensity score-matched cohort.

Over a median follow-up time of 5.00 years (Q1 2.89, Q3 8.38), the risk of DCGL increased sequentially with increasing PRA in transplant-naïve and repeat transplant recipients. The greatest risk was observed in patients with a high PRA (>80%) with prior transplant (adjusted Hazard Ratio (aHR) 1.56, 95% CI 1.47–1.66), followed by a high PRA in transplant-naïve patients (aHR 1.27, 95% CI 1.12–1.43), as shown in Figure 1 (reference category is no prior transplant, 0% PRA). Prior transplant status significantly modified the known association between PRA and DCGL (*p*-value <0.001).

### Secondary analyses

**Figure 2:**
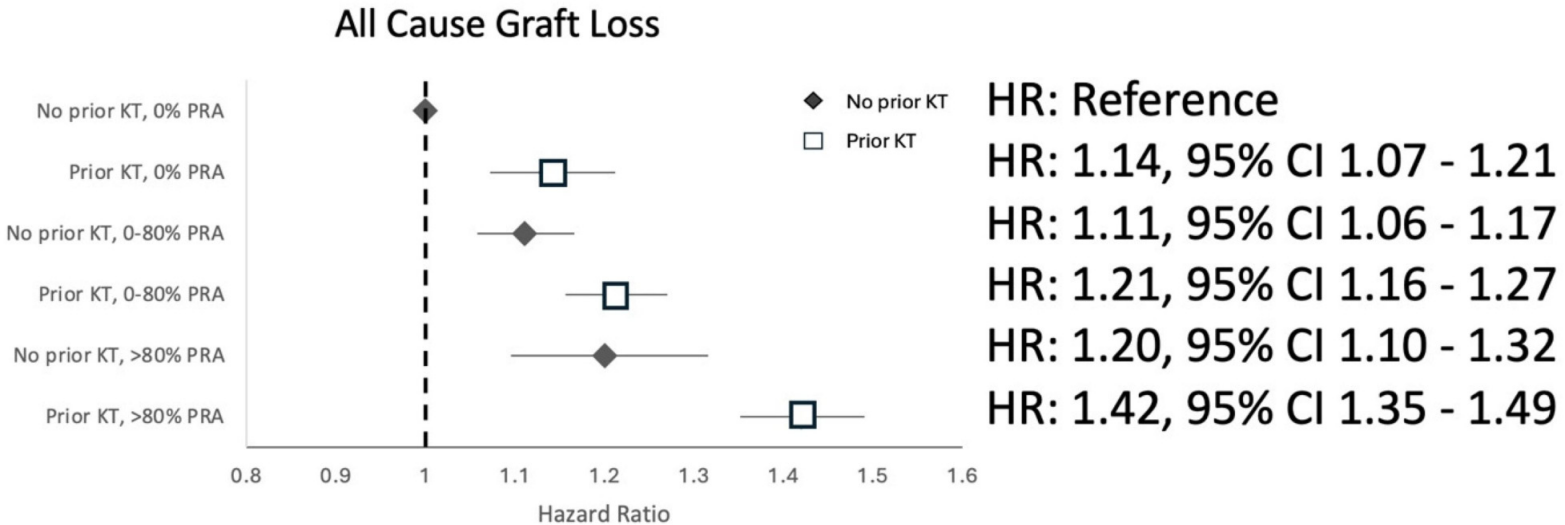
Hazard ratios for all-cause graft loss associated with combined prior transplant and PRA categories in the propensity score-matched cohort.

**Figure 3:**
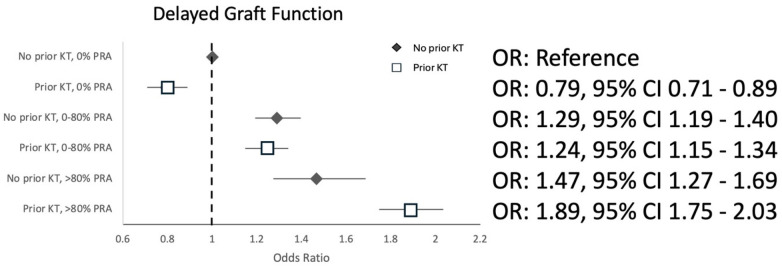
Odds ratios for delayed graft function associated with combined prior transplant and PRA categories in the propensity score-matched cohort.

The risks of ACGL and DGF also increased with increasing PRA, irrespective of prior transplant status. Both outcomes were greatest in patients with a high PRA (>80%) with prior transplant (aHR 1.42, 95% CI 1.35–1.49 for ACGL; aOR 1.89, 95% CI 1.75–2.03 for DGF), followed by a midrange PRA (>0%–80%) with prior transplant for the outcome of ACGL (aHR 1.21, 95% CI 1.16–1.27) and with high PRA (>80%) transplant-naïve patients for the outcome of DGF (adjusted Odds Ratio (aOR) 1.47, 95% CI 1.27–1.69), as shown in Figure 2 (ACGL) and Figure 3 (DGF). A prior transplant significantly modified the known association between PRA and ACGL (*p*-value < 0.001) and PRA and DGF (*p*-value < 0.001 for both).

The number at risk, number of events, proportion of patients with an event, point estimates, exact 95% confidence intervals, *p*-values, and incidence rates per 100 patient-years for all primary and secondary outcomes are summarized in Supplementary Table S2.

### Sensitivity analyses

In a sensitivity analysis, using more granular cut points for PRA did not alter results; the sequentially increased risk for each outcome as PRA increased persisted among both those with and without a prior transplant (Supplementary Figures S2–S4).

After stratifying by PRA grouping (0%, >0%–80%, and >80%), a prior kidney transplant was associated with significantly higher risk of DCGL (aHR 1.23, 95% CI 1.09–1.38), ACGL (aHR 1.18, 95% 1.07–1.29**)**, and DGF (aOR 1.26, 95% CI 1.10–1.45), and these risks were more pronounced in those with high PRA (>80%). ACGL was also increased in patients with a prior transplant at lower PRA levels, whereas DGF risk was reduced in patients with a prior kidney transplant (KT) and PRA <80% (Supplementary Table S3).

In multivariable analyses using the entire study cohort rather than propensity score matching, results were consistent. Those with high PRA (>80%) and a prior transplant were at greatest risk of DCGL (aHR 1.54, 95% CI 1.47–1.62), ACGL (aHR 1.45, 95% 1.40–1.51), and DGF (aOR 1.49, 95% CI 1.40–1.59) (Supplementary Figure S5a–c). In these multivariable models, within each PRA category, patients with a prior transplant had a *significantly* higher risk than transplant-naïve recipients (aside from no difference in unsensitized patients with and without prior transplant for the outcome of DGF).

Given substantial missing data for rejection, we conducted a sensitivity analysis examining the outcome of rejection as captured in the SRTR. The risk of acute rejection was greatest among those with a high PRA (>80%) and a prior transplant, but not significant when patients had a high PRA but were transplant-naïve, acknowledging the small sample size (*n* = 356) and wide confidence intervals (Supplementary Figure S6). Finally, although WIT was significantly longer in those with [36 min (Q1 29, Q3 47)] vs. without [35 min (Q1 28, Q3 46)] a prior transplant (*p*-value 0.004), the differences were clinically negligible (1 min). In the matched cohort, after adjusting for WIT as a continuous variable, results were similar to those of the primary analysis (Supplementary Table S4).

## Discussion

In this study, we demonstrated that after propensity score matching, the risk of both short- (DGF) and long-term (DCGL, ACGL) complications following kidney transplant were highest among sensitized patients with a prior transplant (compared with sensitized, transplant-naïve patients). Importantly, prior transplant status modified the known association between high PRA at transplant and each of DCGL, ACGL, and DGF.

While prior kidney transplant is a major cause of sensitization, the proportion of patients developing detectable anti-HLA antibodies after a prior transplant varies based on center practices regarding continuation of immunosuppression and/or graft nephrectomy (15, 23).

One study of 104 patients indicated sensitization occurred in 70% of patients after a failed transplant (23). Another study of transplant patients in the United States from 2000 to 2018 demonstrated that at the time of first transplant, 87.5% of the patients had a PRA<20%, compared with only 48.2% at the time of re-transplant (22). However, there is limited information on whether *unsensitized* patients with a prior kidney transplant are at increased risk at subsequent transplantation. In one prior study, unsensitized re-transplant recipients had 22% lower graft survival than unsensitized primary transplant recipients; however, this study did not account for differences in case-mix, including potentially greater baseline comorbidity burden among those undergoing repeat as compared with primary transplantation (24). In addition, while this study demonstrated that the mode of sensitization influenced the risk of subsequent graft failure, it focused on patients who were highly sensitized (PRA ≥98%), rather than sensitization status in general (24).

While sensitization status is an established risk factor for adverse posttransplant outcomes including DCGL, ACGL, and DGF (25, 26), the mechanisms of risk associated with prior transplant have long been attributed to an increased risk of HLA antibody formation and subsequent rejection risk (27). In addition, patients undergoing repeat transplantation may be older and more comorbid than those at first transplant, with non-virgin surgical abdomens and longer cumulative dialysis exposure durations. When these factors are not accounted for, risk may also be confounded by differences in case-mix between repeat and transplant-naïve cohorts. For example, as in our current study, Redfield et al. demonstrated that unsensitized re-transplant recipients were at greater risk for graft loss than unsensitized primary transplant recipients; however, although they conducted multivariable analyses, they did not match those with vs. without prior transplant to account for other potentially significant differences in case-mix. In our study, we matched patients based on transplant status, creating comparable populations of patients undergoing first and repeat transplant. Despite this, we demonstrate that after stratifying by PRA at the time of transplant, those with a prior transplant remained at higher risk compared with those undergoing first transplant—for example, a 14% higher risk of ACGL in unsensitized patients with vs. without a prior transplant.

The association between high PRA and DGF is less clear. It is possible that highly sensitized patients may receive more marginal donor kidneys as a means to transplantation (which may explain the greater risk of DGF), though the current study matched on donor factors and still demonstrated a higher DGF risk with greater degrees of sensitization. It has also been proposed that HLA antibodies not detected during crossmatching may induce an acute rejection event that manifests as DGF (28). Other studies have also noted that a previous kidney transplant was potentially an independent predictor of DGF, but a mechanism of action was not provided (29, 30).

The fact that prior transplant status modified the well-established association between PRA and both early and late posttransplant outcomes deserves some consideration. Even in a matched cohort accounting for differences in PRA, patients with a prior transplant were at increased risk compared with transplant-naïve populations. Possible mechanisms include increased surgical risk in patients with prior transplant, even after matching based on differences in recipient age and baseline comorbidity. We hypothesized this may have resulted in prolonged WIT, which is not well captured in the SRTR registry. Longer WIT has been associated with increased risk of death, graft loss (21), and DGF (31). However, in our sensitivity analysis, adjusting for WIT had very little impact on results.

Another important consideration is the potential role of non-HLA antibodies, which are not accounted for in the virtual crossmatch. Evidence suggests that non-HLA antibodies (e.g., angiotensin II type 1 receptor antibodies) are associated with severe and aggressive antibody-mediated rejection and graft failure (32, 33). Other potential non-HLA antibodies have also been noted to impact posttransplant outcomes including those against LG3, perlecan, fibronectin, endothelin-1 type A receptor, and collagen type IV (34–36).

Regardless of the reason—whether on account of unmeasured antibodies, additional surgical risk, or other unknown factors—a prior kidney transplant should be considered a risk factor for worse graft outcomes, irrespective of sensitization status. In most instances, a prior transplant increased the risk of each outcome within a given PRA category. In this study, we demonstrated for the first time that prior transplant status modifies the known association between PRA and early and late graft complications, indicating that the mechanism of risk is not mediated solely through HLA antibody formation. Many centers, including our own, make decisions regarding induction immunosuppression regimens based on recipient PRA at transplant, but not prior transplant status (37), with leukocyte-depleting agents chosen for patients with higher degrees of sensitization. While PRA seems to be a stronger driver of risk than prior transplant status, within each PRA stratum, patients with a prior transplant were at a higher risk for each outcome compared with transplant-naïve recipients, with the exception of unsensitized patients with a prior transplant, who *paradoxically* were at a lower risk of DGF for an unclear reason.

This study is important as it demonstrates for the first time, to our knowledge, that a prior kidney transplant modifies the known association between PRA and graft outcomes. However, there are important limitations that warrant consideration. As with any retrospective analysis, there exists the potential for miscoding or misclassification of data; however, we anticipate that any issues would be distributed at random and unlikely to significantly biased results. Importantly, findings from this observational study indicate an association between prior transplant status and outcomes, but not causality; results must be interpreted with caution and future prospective studies are required to confirm these associations. We lacked data on the cause of sensitization in patients who had not had a prior transplant, or additional sensitizing events in those with prior transplant, to further investigate if there were differences in risk profiles based on the mode of sensitization. It has previously been shown that the mode of sensitization impacts subsequent rejection risk, with prior transplant being the highest risk for subsequent sensitization (24). Notably, we examined the risk of sensitization defined by peak pretransplant PRA—a broad measure of a patient's general immune reactivity. Donor-specific antibodies, which reflect specific antibodies against HLA antigens present on the newly transplanted kidney and are thus associated with a greater rejection risk, were not available in the SRTR. Differences in case-mix between patients with and without a prior kidney transplant have plagued earlier studies. Propensity score matching was performed to achieve similar case-mix among patients with and without a prior kidney transplant; however, this only accounts for variables included in the matching algorithm, and there remains potential for unmeasured confounding influences. In addition, PS matching reduced the sample size, with implications for statistical power when substantifying by PRA and prior transplant status (for example, there were only 1,408 patients included in the >80% PRA transplant-naïve cohort after PS matching). Nevertheless, we still demonstrated significant differences in risk profiles for patients with vs. without a prior kidney transplant at a given PRA category. Another limitation was the significant proportion of missing and incomplete data associated with rejection outcomes, necessitating cautious interpretation of these results, particularly if the data were not missing at random (e.g., those without rejection more likely to be reported as null or missing). This could potentially inflate the risk of rejection in the included cohort. In addition, we lacked longitudinal data on maintenance immunosuppression regimens, treatment strategies for rejection episodes, or reduction of immunosuppression or steroid therapy during the acute phase of illness, all of which may have influenced graft risk over time, particularly among sensitized patients (38).

## Conclusions

In conclusion, this study demonstrated that the risk of both short- (DGF) and long-term (DCGL, ACGL) complications after kidney transplant were highest among sensitized patients with a prior kidney transplant. Moreover, prior kidney transplant status modified the known association between high PRA at transplant and DCGL, ACGL, and DGF. This is important because it could have implications in prescreening for potential kidney transplant recipients and subsequent risk stratification. Future studies should aim to elucidate the mechanisms by which a prior kidney transplant impacts subsequent transplantation, beyond the expected risk associated with increased sensitization.

## Data Availability

Publicly available datasets were analyzed in this study. This data can be found here: https://www.srtr.org/about-the-data/the-srtr-database/.
