# Supplementary material for: The risk of kidney transplant graft loss in sensitized vs. unsensitized patients is modified by prior transplant status
Source: Front Transplant. 2026 Feb 16;5:1712618. doi: 10.3389/frtra.2026.1712618 (PMC12950723; doi:10.3389/frtra.2026.1712618)

**Supplement:**

**Supplemental Table 1**: Baseline characteristics of the propensity score matched population.

| **Variables** | **No Prior Kidney**  **Transplant**  **N=19,330** | **Prior Kidney   Transplant**  **N=19,330** | **Percent Bias**  **(%)** |
| --- | --- | --- | --- |
| **Donor Sex** |  |  |  |
| Male | 10,503 (54.3%) | 10,523 (54.4%) | -0.2 |
| Female | 8,827 (45.7%) | 8,807 (45.6%) |  |
| **Recipient Sex** |  |  |  |
| Male | 11,344 (58.7%) | 11,293 (58.4%) | 0.5 |
| Female | 7,986 (41.3%) | 8,037 (41.2%) |  |
| **Donor Age** | 38 (26, 48) | 38 (25, 48) | 0.5 |
| **Recipient Age** | 45 (33, 55) | 45 (35, 54) | 3.5 |
| **Donor Race** |  |  |  |
| Black | 2,310 (12.0%) | 2,266 (11.7%) | -1.6 |
| White | 16,388 (84.8%) | 16,485 (85.3%) |  |
| Other | 632 (3.3%) | 579 (3.0%) |  |
| **Recipient Race** |  |  |  |
| Black | 4,528 (23.4%) | 4,315 (22.3%) | -2.0 |
| White | 13,874 (71.8%) | 14,096 (72.9%) |  |
| Other | 928 (4.8%) | 919 (4.8%) |  |
| **Preemptive** |  |  |  |
| Yes | 2,971 (15.4%) | 2,867 (14.8%) | 1.5 |
| No | 16,359 (84.6%) | 16,463 (85.2%) |  |
| **HLA** |  |  |  |
| 0 | 2,704 (14.0%) | 2,932 (15.2%) | -2.6 |
| 1 | 941 (4.9%) | 1,002 (5.2%) |  |
| 2 | 1,983 (10.3%) | 1,834 (9.5%) |  |
| 3 | 3,695 (19.1%) | 3,542 (18.3%) |  |
| 4 | 4,064 (21.0%) | 4,182 (21.6%) |  |
| 5 | 4,053 (21.0%) | 4,088 (21.2%) |  |
| 6 | 1,890 (9.8%) | 1,750 (9.1%) |  |
| **Recipient Comorbidities** |  |  |  |
| Diabetes | 4,109 (21.3%) | 4,131 (21.4%) | 0.3 |
| Hypertension | 16,492 (85.3%) | 16,486 (85.3%) | -0.1 |
| PVD | 786 (4.1%) | 795 (4.1%) | 0.2 |
| CAD | 1,320 (6.8%) | 1,379 (7.1%) | 1.1 |
| **Cause of ESKD** |  |  |  |
| Diabetes | 3,392 (17.6%) | 2,654 (13.7%) | 2.3 |
| GN | 6,673 (34.5%) | 7,616 (39.4%) |  |
| PCKD | 1,897 (9.8%) | 1,266 (6.6%) |  |
| Hypertension | 4,499 (23.3%) | 3,513 (18.2%) |  |
| Hereditary | 517 (2.7%) | 906 (4.7%) |  |
| Drugs | 351 (1.8%) | 220 (1.1%) |  |
| Other | 2,001 (10.4%) | 3,155 (16.3%) |  |
| **Donor Type** |  |  |  |
| Deceased | 13,036 (67.4%) | 13,381 (69.2%) | -3.8 |
| Living | 6,294 (32.6%) | 5,949 (30.8%) |  |
| **Donor-Recipient Weight Ratio** |  |  |  |
| < 10 kg | 6,624 (34.3%) | 5,887 (30.5%) | -0.0 |
| 10 to 30 kg | 4,196 (21.7%) | 4,756 (24.6%) |  |
| > 30 kg | 1,995 (10.3%) | 2,751 (14.2%) |  |
| -10 to -30 kg | 4,129 (21.2%) | 3,893 (20.1%) |  |
| < -30 kg | 2,386 (12.3%) | 2,043 (10.6%) |  |
| **Peak PRA*** | 0(0,16) | 58(6,92) | - |
| 0 | 16,013 (82.8%) | 8,698 (45.0%) |  |
| 0-80% | 1,822 (9.4) | 5,511 (28.5%) |  |
| >80%  **Year of Transplant** | 688 (3.6%)  2008(2004,2011) | 3,877 (20.1%)  2007(2004,2011) | -1.4 |

*Abbreviations:  Human leukocyte antigen (HLA); peripheral vascular disease (PVD); coronary artery disease (CAD); end-stage kidney disease (ESKD); glomerulonephritis (GN); polycystic kidney disease (PCKD); body mass index (BMI); panel reactive antibody (PRA)*

*PRA was not included in the propensity score matching

**Supplemental Table 2:** The number at risk, number of events, proportion of patients with an event, point estimate and exact 95% confidence intervals, p-values, and incidence rates per 100-person years for all primary and secondary outcomes.

|  | **Number** | **Event** | **Proportion with Event** | **Incidence Rate per 100-person years (95% CI)** | **Risk Ratio (95% CI)** | **p-value** |
| --- | --- | --- | --- | --- | --- | --- |
|  | **Death Censored Graft Loss** | | | | | |
| No Prior KT, 0% PRA | 10,084 | 1971 | 19.5% | 3.2 (3.1-3.4) | Reference | - |
| No Prior KT, 0-80% PRA | 7838 | 1751 | 22.3% | 3.8 (3.6-3.9) | aHR 1.16 (1.09-1.24) | <0.001 |
| No Prior KT, >80% PRA | 1408 | 314 | 22.3% | 4.1 (3.6-4.5) | aHR 1.27 (1.12-1.43) | <0.001 |
| Prior KT, 0% PRA | 3653 | 750 | 20.5% | 3.4 (3.1-3.6) | aHR 1.04 (0.95-1.13) | 0.412 |
| Prior KT, 0-80% PRA | 8426 | 2015 | 23.9% | 4.0 (3.8-4.2) | aHR 1.23 (1.16-1.31) | <0.001 |
| Prior KT, >80% PRA | 7251 | 1873 | 25.8% | 5.0 (4.8-5.2) | aHR 1.56 (1.47-1.66) | <0.001 |
|  | **All Cause Graft Loss** | | | | | |
| No Prior KT, 0% PRA | 10,084 | 3500 | 34.7% | 5.7 (5.6-5.9) | Reference | - |
| No Prior KT, 0-80% PRA | 7838 | 2977 | 38.0% | 6.4 (6.2-6.6) | aHR 1.11 (1.06-1.17) | <0.001 |
| No Prior KT, >80% PRA | 1408 | 526 | 37.4% | 6.8 (6.2-7.4) | aHR 1.20 (1.10-1.32) | <0.001 |
| Prior KT, 0% PRA | 3653 | 1469 | 40.2% | 6.6 (6.3-6.9) | aHR 1.14 (1.07-1.21) | <0.001 |
| Prior KT, 0-80% PRA | 8426 | 3529 | 41.9% | 7.0 (6.8-7.2) | aHR 1.21 (1.16-1.27) | <0.001 |
| Prior KT, >80% PRA | 7251 | 3003 | 41.4% | 8.0 (7.7-8.3) | aHR 1.42 (1.35-1.49) | <0.001 |
|  | **Delayed Graft Function** | | | | | |
| No Prior KT, 0% PRA | 10,084 | 1516 | 15.0% |  | Reference |  |
| No Prior KT, 0-80% PRA | 7838 | 1457 | 18.6% |  | aOR 1.29 (1.19-1.40) | <0.001 |
| No Prior KT, >80% PRA | 1408 | 290 | 20.6% |  | aOR 1.47 (1.27-1.69) | <0.001 |
| Prior KT, 0% PRA | 3653 | 450 | 12.3% |  | aOR 0.79 (0.71-0.89) | <0.001 |
| Prior KT, 0-80% PRA | 8426 | 1517 | 18.0% |  | aOR 1.24 (1.15-1.34) | <0.001 |
| Prior KT, >80% PRA | 7251 | 1814 | 25.0% |  | aOR 1.89 (1.75-2.03) | <0.001 |

**Supplemental Table 3**:  The adjusted risk of each of death-censored graft loss, all-cause graft loss, and delayed graft function associated with a prior kidney transplant (versus no prior transplant) after stratifying by PRA.

|  | **PRA 0%** | **PRA >0-80%** | **PRA >80%** |
| --- | --- | --- | --- |
| **DCGL (HR)** | 1.03 (0.95-1.13) | 1.03 (0.96-1.10) | 1.23 (1.09-1.38) |
| **ACGL (HR)** | 1.14 (1.07- 1.21) | 1.09 (1.03- 1.14) | 1.18 (1.07-1.29) |
| **DGF (OR)** | 0.79 (0.71-0.89) | 0.92 (0.84-1.00) | 1.26 (1.10-1.45) |

*All models are also adjusted for PRA as a continuous variable.

**Supplemental Table 4**: Adjusted hazard ratio for death censored graft loss associated and all cause graft loss, and adjusted odds ratio for delayed graft function with combined prior transplant and PRA cut points in the propensity score matched cohort adjusting for warm ischemia time.

|  | **DCGL** | **ACGL** | **DGF** |
| --- | --- | --- | --- |
| **No prior KT, 0% PRA** | Ref | Ref | Ref |
| Prior KT, 0% PRA | 1.01 (0.91-1.12) | 1.16 (1.10-1.22) | 0.76 (0.66-0.87) |
| No prior KT, 0-80% PRA | 1.10 (1.01-1.19) | 1.03 (1.01-1.04) | 1.25 (1.14-1.38) |
| Prior KT, 0-80% PRA | 1.14 (1.06-1.23) | 1.18 (1.14-1.22) | 1.22 (1.11-1.34) |
| No prior KT, >80% PRA | 1.17 (1.01-1.36) | 1.19 (1.15-1.23) | 1.34 (1.14-1.59) |
| Prior KT, >80% PRA | 1.46 (1.35-1.58) | 1.45 (1.40-1.51) | 1.83 (1.67-2.01) |

**Supplemental Figure 1:** Flow diagram for cohort derivation.


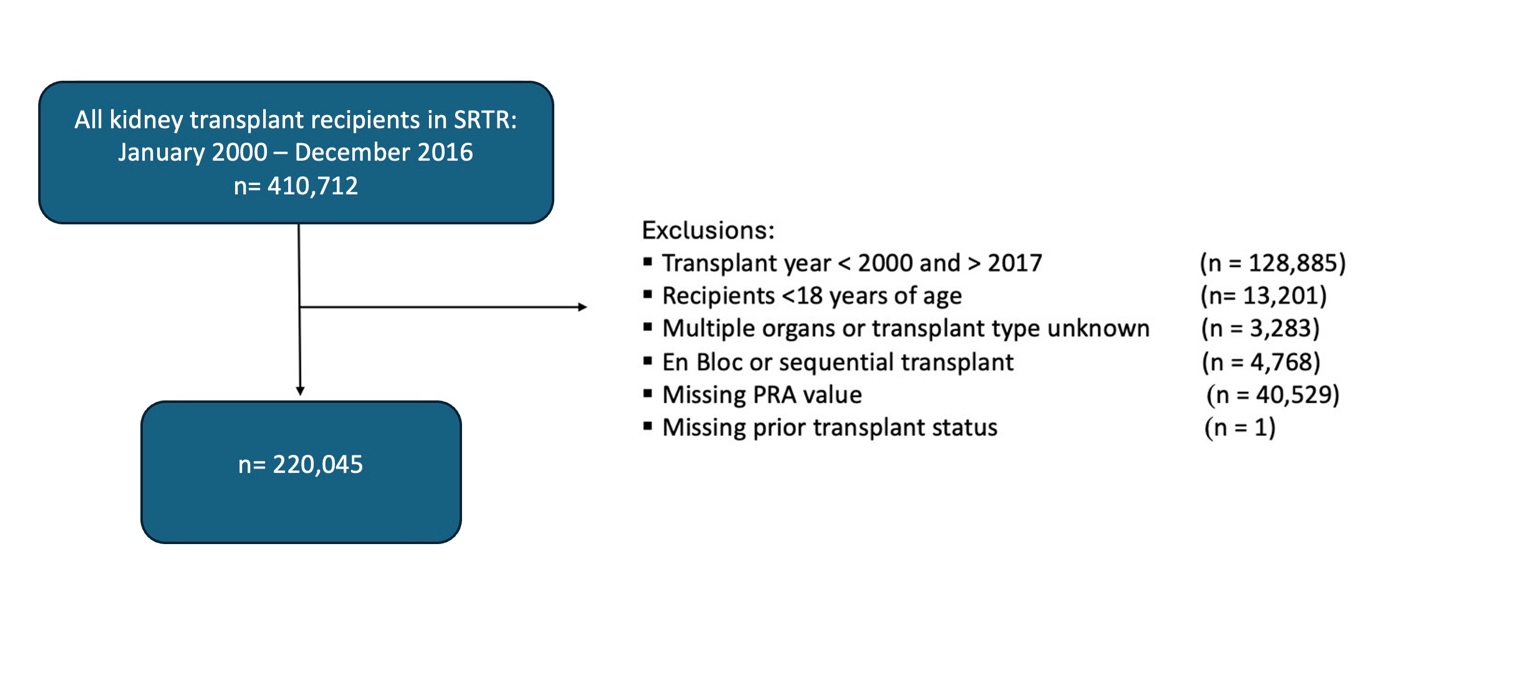


**Supplemental Figure 2:** Hazard ratios for death censored graft loss associated with combined prior transplant and more granular cut points for PRA categories in the propensity score matched cohort.


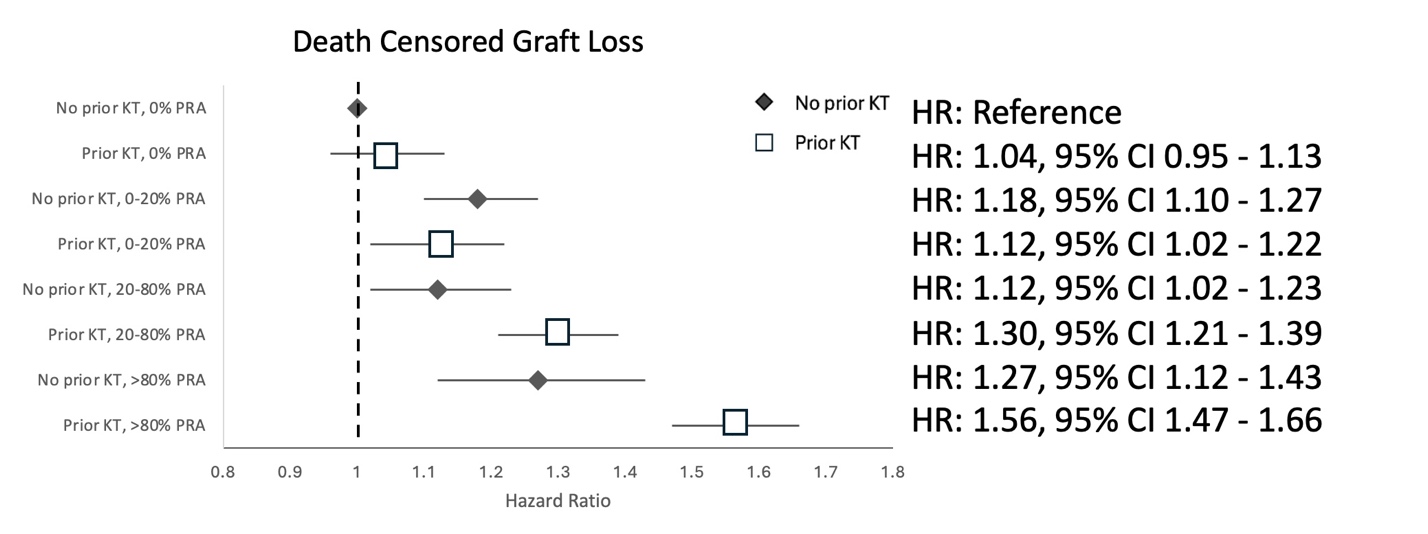


**Supplemental Figure 3:** Hazard ratios for all cause graft loss associated with combined prior transplant and more granular cut points for PRA categories in the propensity score matched cohort.


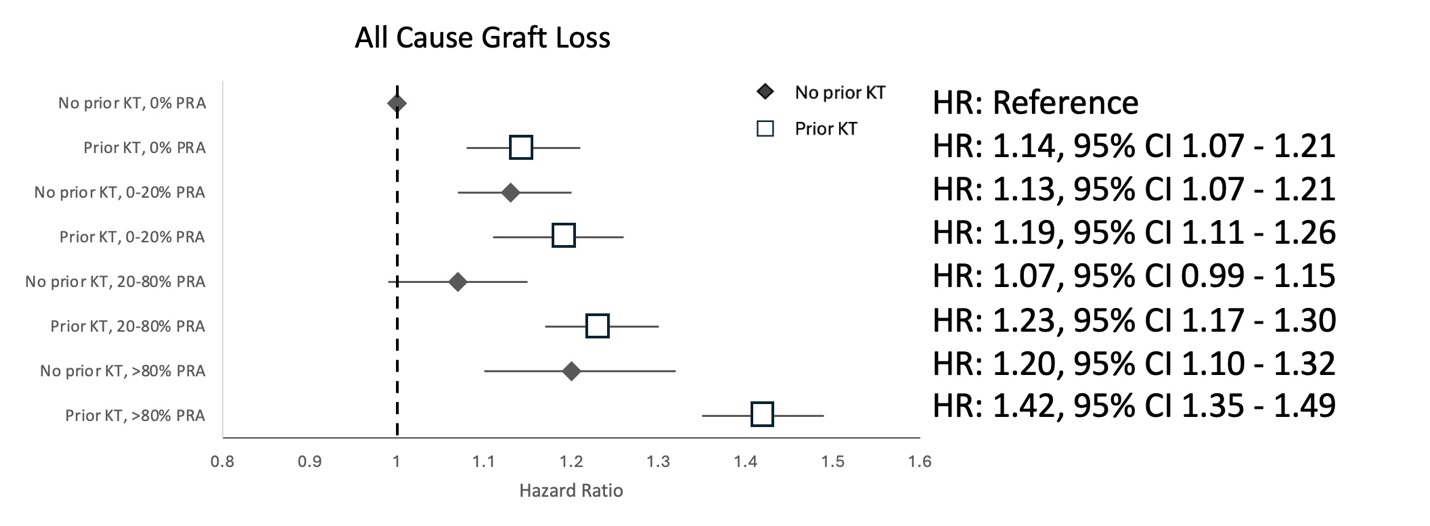


**Supplemental Figure 4**: Odds ratios for delayed graft function associated with combined prior transplant and more granular cut points for PRA categories in the propensity score matched cohort.


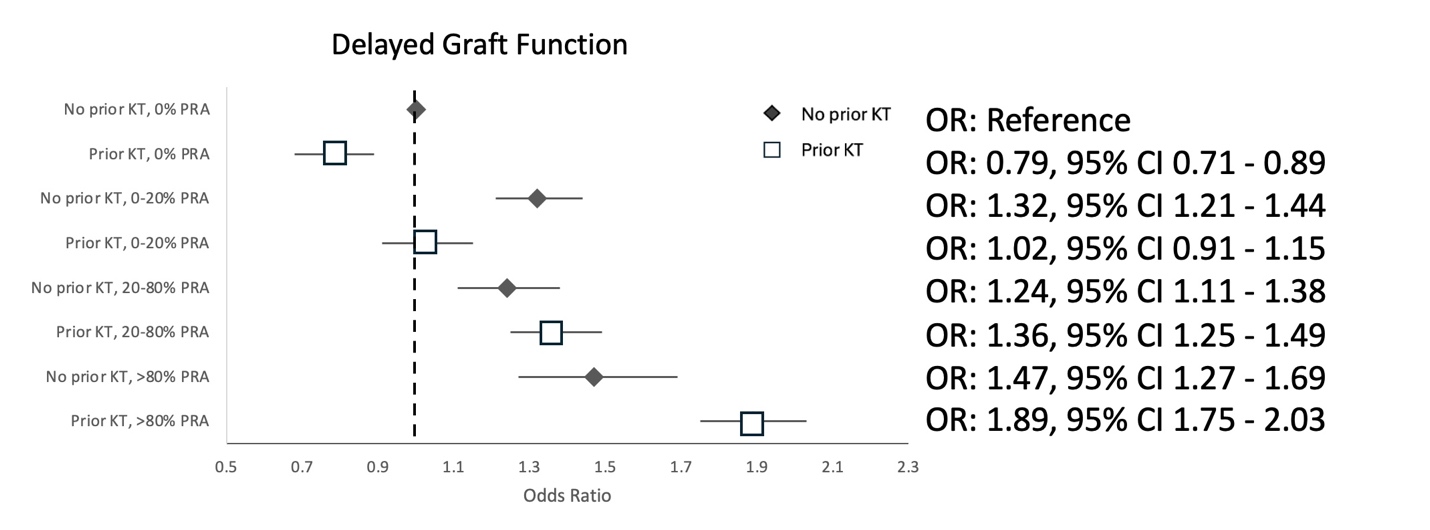


**Supplemental Figure 5a**: Multivariable model of hazard ratios for death censored graft loss associated with combined prior transplant and PRA categories in entire cohort.


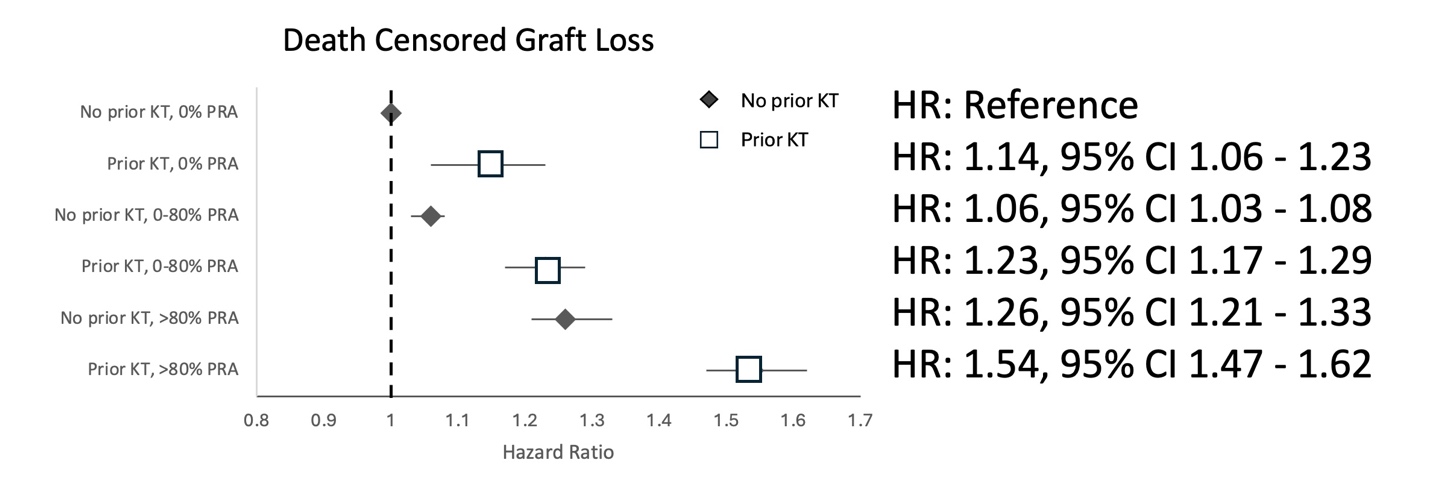


**Supplemental Figure 5b:** Multivariable model of hazard ratios for all cause graft loss associated with combined prior transplant and PRA categories in entire cohort.

**
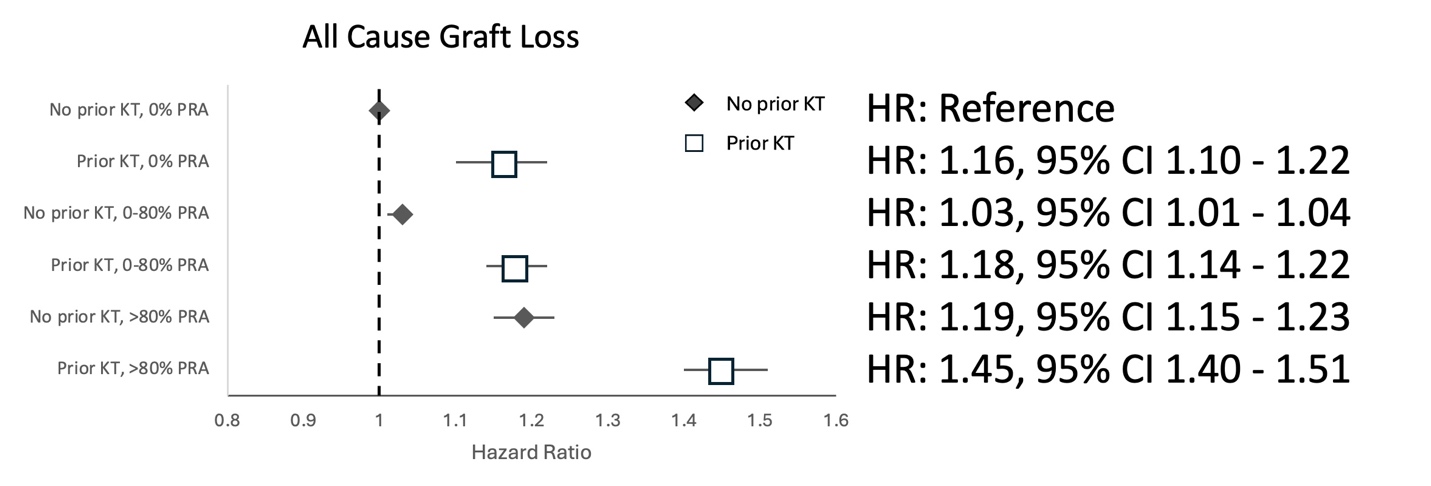
**

**Supplemental Figure 5c:** Multivariable model of odds ratios for delayed graft function associated with combined prior transplant and PRA categories in entire cohort.


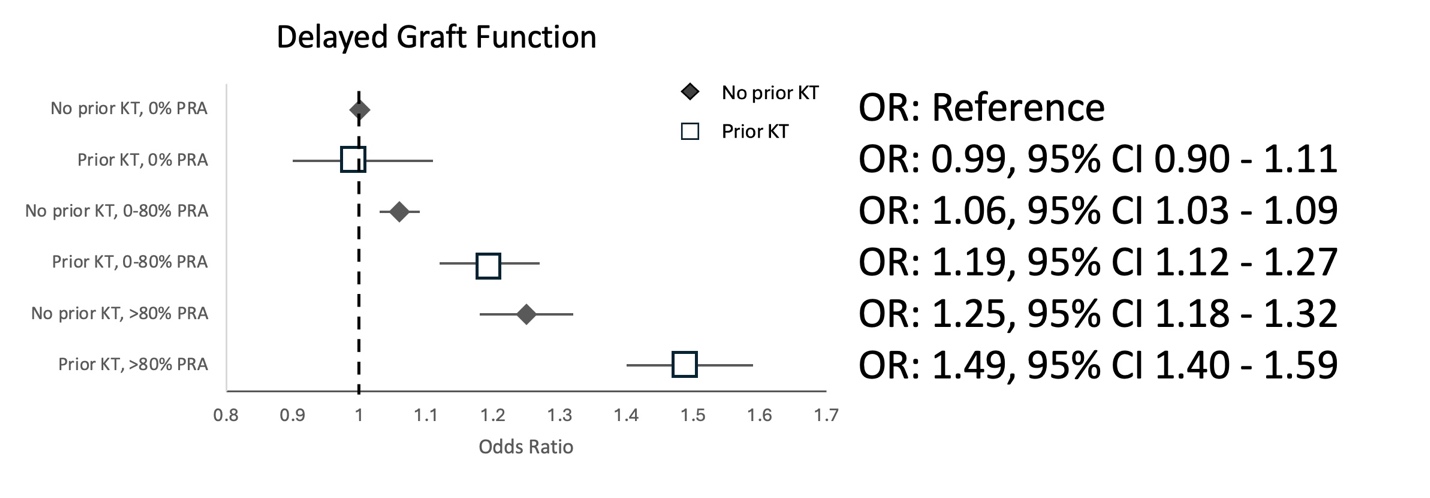


**Supplemental Figure 6:** Odds of transplant rejection using a logistic regression model in the PS matched cohort amongst those with applicable data.


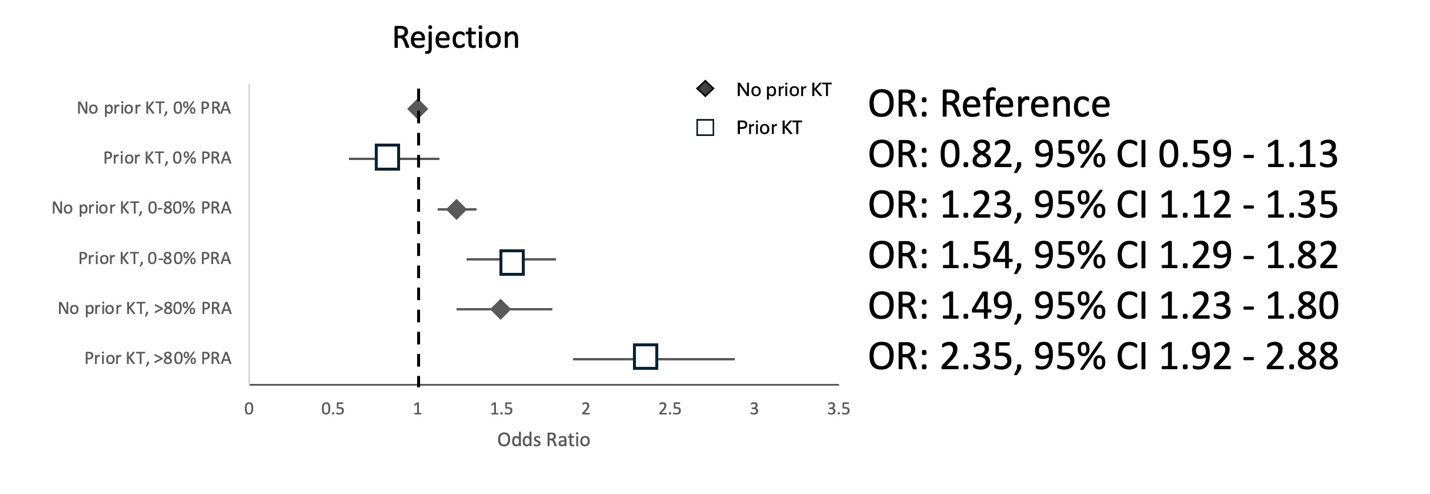

Supplement: Supplementary file 1 [file Table1.docx]
